# Life course trajectories of maternal cardiovascular disease risk factors by obstetric history: a UK cohort study using electronic health records

**DOI:** 10.1186/s12916-025-03937-y

**Published:** 2025-02-14

**Authors:** Kate Birnie, Laura D. Howe, Timothy Jones, Paul Madley-Dowd, Florence Z. Martin, Harriet Forbes, Maria Theresa Redaniel, Rosie Cornish, Maria C. Magnus, Neil M. Davies, Kate Tilling, Alun D. Hughes, Deborah A. Lawlor, Abigail Fraser

**Affiliations:** 1 MRC Integrative Epidemiology Unitat the , University of Bristol, Bristol, UK; 2 Bristol Medical School, Population Health Sciences, University of Bristol, Bristol, UK; 3 The National Institute for Health Research and Applied Research Collaboration West (NIHR ARC West), University Hospitals Bristol and Weston NHS Foundation Trust, Bristol, UK; 4 Bristol Medical School, Translational Health Sciences, University of Bristol, Bristol, UK; 5 NIHR Bristol Biomedical Research Centre, University Hospitals Bristol and Weston NHS Foundation Trust and University of Bristol, Bristol, UK; 6 Faculty of Epidemiology and Population HealthandDepartment of Non-Communicable Disease EpidemiologySchool of Hygiene and Tropical Medicine, London, UK; 7 National Cancer Registry Ireland, Cork, Ireland; 8 Centre for Fertility and Health, Norwegian Institute of Public Health, Oslo, Norway; 9 Division of Psychiatry, University College London, London, UK; 10 Department of Statistical Sciences, University College London, London, UK; 11 K.G. Jebsen Center for Genetic Epidemiology, Department of Public Health and Nursing, Norwegian University of Science and Technology, Trondheim, Norway; 12 MRC Unit for Lifelong Health and Ageing at University College London, London, UK; 13 Department of Population Science and Experimental Medicine, Institute of Cardiovascular Science, University College London, London, UK

**Keywords:** Adverse pregnancy outcomes, Preeclampsia, Gestational hypertension, Gestational diabetes, Cardiovascular risk factors

## Abstract

**Background:**

Women who experience adverse pregnancy outcomes (APOs; gestational hypertension, preeclampsia (PE), gestational diabetes (GD), preterm birth (PTB), small or large for gestational age, miscarriage, multiple miscarriages, stillbirth, and offspring with major congenital anomalies) have increased risk of developing cardiovascular disease (CVD). We aimed to compare cardiometabolic health trajectories across the life course between women with and without APOs.

**Methods:**

We studied 187,186 women with a registered pregnancy in the UK Clinical Practice Research Datalink (CPRD) GOLD linked to Hospital Episode Statistics. Fractional polynomial multilevel models were used to compare trajectories of cardiometabolic risk factors (body mass index [BMI], blood pressure [BP], cholesterol, and glucose) between women with and without a history of APOs (individual APOs in any pregnancy and number of APOs). We explored two underlying time axes: (1) time relative to first pregnancy (from 10 years before first pregnancy to 15 years after) and (2) age. Models controlled for age at first pregnancy, residential area deprivation, non-singleton pregnancy, parity, smoking status, ethnicity, and medications use.

**Results:**

Women with a history of PE, gestational hypertension, or GD had higher BMI, BP, and glucose 10 years before first pregnancy compared to women without these APOs. These differences persisted 15 years post-first pregnancy. Women with a history of GD had a steeper post-partum rise in glucose. Women who experienced multiple (3 +) miscarriage, stillbirth, and/or medically indicated PTB had higher BP and BMI before and after pregnancy, with BP trajectories converging 15 years after first pregnancy. Women who experienced multiple APOs had the most adverse measurements across all cardiometabolic risk factors, with more unfavourable mean levels with each additional APO. There was little difference in cardiometabolic trajectories between women with and without a history of 1 or 2 miscarriages or congenital anomalies.

**Conclusions:**

Women with APOs had adverse cardiometabolic profiles before first pregnancy, persisting up to 15 years post-pregnancy. Findings highlight the potential for targeted public health interventions to promote good cardiometabolic health in young adults transitioning from contraceptive use to planning pregnancies. APOs may identify young women who could benefit from monitoring CVD risk factors and interventions to improve cardiometabolic health.

**Supplementary Information:**

The online version contains supplementary material available at 10.1186/s12916-025-03937-y.

## Background

Pregnancy places considerable demands on the maternal cardiovascular system. It is well established that women with a history of adverse pregnancy outcomes (APOs), including gestational hypertension, preeclampsia (PE), gestational diabetes (GD), and preterm birth (PTB), are more likely to have cardiovascular disease (CVD) later in life compared to parous women without a history of APOs [1–7]. Emerging evidence suggests that women who develop APOs already have more adverse levels of cardiometabolic risk factors before pregnancy, and that pre-existing differences in CVD risk factors between women with and without APOs remained fairly constant across the life course [8–10]. This suggests that APOs may ‘unmask’ women with a pre-existing (prior to pregnancy) propensity for CVD and that some APOs, such as PE, are ‘cardiovascular complications of pregnancy’ [11]. Less is known about life course cardiometabolic health of women who experience miscarriage, multiple miscarriage, stillbirth, or who have offspring with major congenital anomalies, but recent studies suggest that women experiencing these APOs have a higher risk of CVD later in life [12, 13]. Furthermore, few prospective studies have looked at the association between experiencing multiple APOs and a woman’s long-term CVD risk. Findings from a Swedish registry-based national cohort study showed that women who experienced any of five major APOs (PTB, small for gestational age [SGA], PE, other hypertensive disorders of pregnancy, and GD) showed an increased risk of subsequent ischemic heart disease and women who experienced multiple APOs showed further increases in risk [2]. A population-based prospective study of Norwegian registry data found that risk of dying from atherosclerotic CVD increased with the number of pregnancies complicated by APOs (PTB, PE, placental abruption, perinatal death, low birth weight) in a strong dose–response relationship [14].

Here, we describe women’s cardiometabolic health trajectories from before first pregnancy and until around 50 years of age according to history of APOs among women with a registered pregnancy in the UK Clinical Practice Research Datalink (CPRD). We examined cardiometabolic health trajectories by the presence or absence of individual APOs (gestational hypertension, PE, GD, PTB, SGA, large for gestational age (LGA), miscarriage, multiple miscarriages, stillbirth, and offspring with major congenital anomalies) and also according to the number of APOs experienced across all pregnancies. A wide range of cardiometabolic health measures were evaluated: systolic blood pressure (BP), diastolic BP, body mass index (BMI), total cholesterol, high-density lipoprotein (HDL) cholesterol, and glucose.

## Methods

### Data source

This electronic health record study used linked primary and secondary care data for people in England registered with the National Health Service. CPRD GOLD contains pseudonymised health data on over 11.3 million people (defined as anyone who has at least one record of health care provision in CPRD GOLD), including data on clinical consultations, diagnoses, therapies, referrals, and tests. It broadly represents the UK general population’s age, sex, and ethnicity [15]. We used the CPRD Pregnancy Register to identify women who had experienced at least one pregnancy between 1 January 1997 and 31 December 2019. The methods for creating the CPRD Pregnancy Register are described in detail elsewhere, and validation studies show good concordance with hospital delivery data [16]. The CPRD Pregnancy Register is based on an algorithm that identifies pregnancies using antenatal, birth, and postnatal care administrative codes in both mother and child primary care records (where available). Using a patient identifier, we linked to inpatients hospitalisations data from Hospital Episode Statistics (HES) Admitted Patient Care [17] for information on maternity records; this provided information on birth weight and allowed us to recode some pregnancy outcomes coded as unknown in the CPRD Pregnancy Register to known pregnancy outcomes based on the HES data [18]. Using a patient identifier, data were linked to Townsend area level deprivation scores for each patient based on their home address postcode [19]. Data were also linked to a CPRD-developed probabilistic mother-baby link algorithm [20], which links mother-baby pairs within the CPRD GOLD database; this allowed us to identify major congenital anomalies in offspring records (where available).

### Study population

We included all women whose first pregnancy was recorded in CPRD GOLD from 1997 to 2019. We excluded women not registered for at least 3 months with a General Practice (provider of primary care) that met the criteria for being ‘up to standard’ (UTS) before their pregnancy start date, to ensure accurate recordings of their pregnancies. The UTS date refers to a practice-level quality metric based on the continuity of recordings and the number of recorded deaths [15]. We also excluded women who were not eligible for linkage to HES because they were based outside of England, or where the GP practice or individual opted out of linkage. We did not exclude women with pre-existing medical conditions, such as hypertension prior to pregnancy, from analyses.

### Cardiometabolic risk factors

From the primary care data, we extracted all available recorded repeated measures for each woman for the following cardiometabolic risk factors: BMI (kg/m^2^), systolic BP (mmHg), diastolic BP (mmHg), total cholesterol (mmol/L), high-density lipoprotein (HDL) cholesterol (mmol/L), and glucose (fasting and non-fasting; mmol/L). We excluded cardiometabolic measurements taken during pregnancy. We extracted measures from when records began, including prior to each woman’s UTS date, to maximise the number of data points in the analysis. A woman needed at least one recorded cardiometabolic risk factor to be included in each analysis. In the analysis of glucose, we controlled for fasting status.

### APOs

Code lists for APOs were developed by using published lists [21], consulting with clinical experts, and searching the medical and product code dictionaries for Read and ICD-10 codes (available at https://github.com/123KB/Cardiometabolic-trajectories-by-APOs). We examined the following APOs, in any pregnancy experienced by each woman (i.e. not just their first pregnancy), compared to women who did not experience this APO: gestational hypertension; PE; GD; PTB (defined by HES maternity records of < 37 completed weeks gestation, and classified as spontaneous onset or medically indicated, medically indicated PTB was where labour was induced or delivery was initiated by caesarean section prior to onset of labour); SGA and LGA (defined by birth weight and gestational length from HES maternity records: we used cut-offs of < 10th centile for SGA age and > 90th centile for LGA; women with LGA were excluded from the comparator group in the SGA analysis, and vice versa); miscarriage (recorded as the outcome in the Pregnancy Register, or from HES records if the outcome was recorded as unknown in the Pregnancy Register); multiple miscarriages (defined as 3 or more miscarriages across pregnancies, not necessarily consecutive); stillbirth (recorded in the Pregnancy Register, or from HES records if the outcome was recorded as unknown in the Pregnancy Register); offspring with major congenital anomalies (defined via linked baby records). We used a published code list to identify major congenital anomalies [22], which had been developed following guidelines published by the ‘European Surveillance of Congenital Anomalies’ network (EUROCAT) [23]. Women who experienced pregnancies where the offspring had major congenital anomalies were compared to women where there was no evidence of a congenital anomaly, or evidence of minor congenital anomalies. The majority (> 90%) of major congenital anomalies were identified antenatally and we could not identify congenital anomalies in pregnancies that were terminated. In addition, we compared women who had experienced any APO with women who had experienced only healthy pregnancies across their lifetime. For further details on APO coding see Additional file 1: Text S1.

### Post hoc* categorisation of APOs*

In the comparison between women with any APO to women with only healthy pregnancies (defined as a ‘live birth’ from the Pregnancy Register, without any APO), any APO included gestational hypertension, PE, GD, PTB, SGA, LGA, multiple miscarriages, and stillbirth. Women who experienced 1 or 2 miscarriages and had offspring with major congenital anomalies were not included in the definition of any APO, due to there being little difference in cardiometabolic trajectories between women with and without these conditions (see ‘Results’). The number of APOs (gestational hypertension, PE, GD, PTB, SGA, LGA, multiple miscarriages, or stillbirth) experienced across all of a woman’s pregnancies (including if multiple APOs were experienced in a single pregnancy) was categorised as 0, 1, 2, and 3 + . For women with PE complicated by SGA or medically indicated PTB, each condition was counted separately. For example, a pregnancy complicated by both PE and SGA would contribute two APOs to the total count for that woman. Similarly, for women with GD complicated by LGA, both conditions were counted separately.

### Covariates and potential confounders

Information on age at first pregnancy (years), whether women had ever experienced a non-singleton pregnancy (no/yes), and parity (total number of pregnancies) was obtained from the Pregnancy Register. Townsend residential area level deprivation quintile (an index of material deprivation and disadvantage; 1 [least deprived], 2, 3, 4, 5 [most deprived]) was acquired by data linkage based on patient postcode. Information on whether women had ever smoked prior to the start of their first pregnancy was obtained from the primary care medical codes, and were otherwise coded as never-smokers. Ethnicity data (White and non-White) was obtained from HES. Medicine usage was obtained from primary care records (prescriptions of antihypertensives, hypoglycaemics, statins, and other cholesterol lowering medicines).

### Statistical analysis

We compared the characteristics of the cohort and compare women who were in the Pregnancy Register, but not eligible for inclusion in the study to assess for selection bias. We described the variation in the number of repeat measures for each trajectory. We used counts (percentages) to describe categorical variables, means (standard deviations [SD]) for approximately normally distributed continuous variables, and medians (interquartile ranges [IQR]) for continuous variables with right-skewed distributions. For the trajectories, we provided the median number of repeat outcome measurements, IQR, and full range.

Multilevel models with random intercepts were used to estimate trajectories of each cardiometabolic risk factor with differing time axes: (1) over time relative to first pregnancy and (2) over age, for women with and without each APO. We only included random intercepts (and not additionally random slopes) because this was a more parsimonious model. We used multilevel fractional polynomials [24] to allow for non-linear trajectories, choosing the best-fitting model with 1 or 2 degrees (see Additional file 1: Text S2 for more details). In the analysis with time on the x-axis, three-way interaction terms between each of the polynomial terms, the APO and whether the cardiometabolic measure was before or after the first pregnancy, were used to examine whether cardiometabolic trajectories vary between women with and without an APO. Models controlled for age at first pregnancy, Townsend residential area deprivation score quintile, whether the women had experienced non-singleton pregnancies, the total number of pregnancies for each woman, smoking status, ethnicity, and use of medicines. We plotted predicted mean levels of cardiometabolic risk factors and 95% confidence intervals (CIs) over age or time for women with and without each APO, with average levels of the included covariates. All analyses were performed in Stata 18.

In the main analysis, we included everyone who had at least one cardiometabolic outcome measure and carried out a sensitivity analysis on women with at least one pre- and at least one post-pregnancy measure. We also carried out a sensitivity analysis by fitting a more complex model with random intercepts and random slopes for systolic BP (the cardiometabolic outcome with the most measures per person).

## Results

### Characteristics of the study population and comparison with women who were not included

**Fig. 1:**
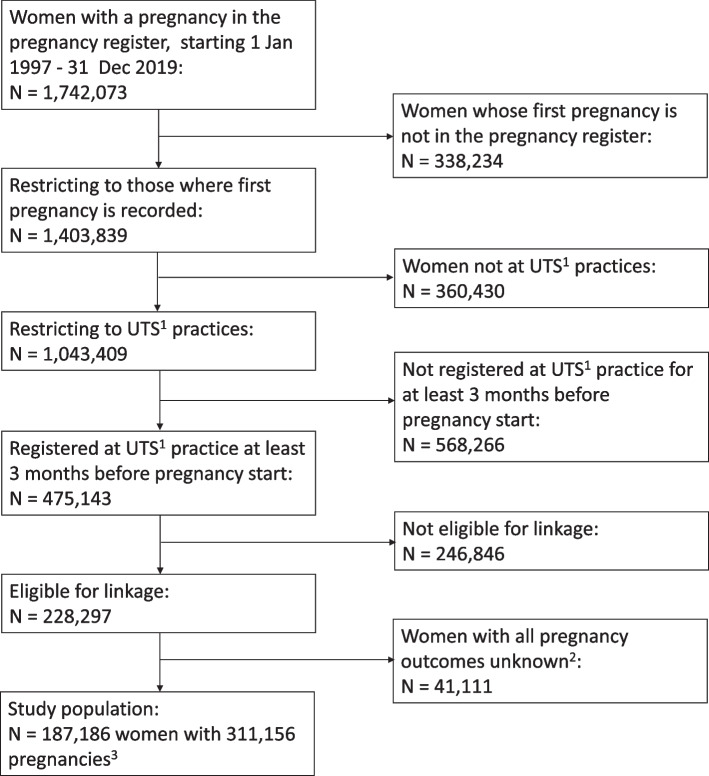
Flow chart of women included in the cohort. ^1^UTS means the practice is ‘up to standard’, a practice-level quality metric. ^2^These unknown pregnancy outcomes were not resolved using HES data. The number in this box includes women with ongoing pregnancies at the time of data extraction, estimated to be around 1000. ^3^After data cleaning, including removing duplicate pregnancy records

The study cohort included 187,186 women, with 311,156 pregnancies, registered in CPRD GOLD. The derivation of the cohort is illustrated in Fig. 1. Women had an average of 5 years’ registration with their GP practice prior to first pregnancy (median 5.1, IQR 1.8, 14.9 years; Additional file 1: Table S1). Average registration times before first pregnancy were relatively stable across calendar years, whereas data collection times after first pregnancy decreased over the years, reflecting a shorter follow-up period for more recent pregnancies. Characteristics of included women are shown in Table 1; women had a mean age at first pregnancy of 27.1 years (SD 6.6). Most women (86.5%) were of White ethnicity; 21.2% were in the least deprived Townsend quintile, and 16% were in the most deprived Townsend quintile. Gestational hypertension was experienced by 9.4% of women across all of their pregnancies, PE by 4.0%, GD by 4.0%, spontaneous PTB by 5.6%, medically indicated PTB by 3.1%, SGA by 12.4%, LGA by 11.7%, any miscarriage by 18.6%, multiple miscarriages by 0.6%, stillbirth by 0.7%, and delivering offspring with major congenital anomalies by 3.1%. Compared to all women in the CPRD GOLD Pregnancy Register between 1997 and 2019, the women included in the cohort are more likely to be of White ethnicity (86.5% vs. 81.2%) and in the least deprived Townsend quintile (21.1% vs. 18.8%) (Additional file 1: Table S2).

**Table 1:** Characteristics of the study population

Characteristic	Level	*N* (%) or mean (SD)
Age at first pregnancy	Years	27.1 (6.6)
Experienced multiple pregnancy	Yes	2020 (1.1)
Parity	1	77,951 (41.6)
	2	52,643 (28.1)
	3	28,116 (15.0)
	4	14,994 (8.0)
	5 +	13,482 (7.2)
Ethnicity^a^	White	156,968 (86.5)
	Non-White	18,504 (10.2)
	Unknown	6027 (3.3)
Patient level Townsend score quintile^b^	1 (least deprived)	39,641 (21.2)
	2	38,055 (20.4)
	3	39,086 (20.9)
	4	40,357 (21.6)
	5 (most deprived)	29,848 (16.0)
Smoking status	Never	102,906 (55.0)
	Ever	84,280 (45.0)
Antihypertensives	Ever	11,069 (5.9)
Cholesterol lowering medicines	Ever	619 (0.3)
Hypoglycaemics	Ever	1957 (1.0)
Gestational hypertension	Yes	17,676 (9.4)
PE	Yes	7521 (4.0)
GD	Yes	7450 (4.0)
Preterm delivery^c^	Spontaneous onset	5739 (5.6)
	Medically indicated	3178 (3.1)
SGA^c^	Yes	12,646 (12.4)
LGA^c^	Yes	11,977 (11.7)
Multiple miscarriage	Yes	1117 (0.6)
Stillbirth	Yes	1297 (0.7)
Any APO^d^	Yes, experienced APO	56,190 (40.2)
	No, healthy pregnancy	83,471 (59.8)
Number of APOs	0	134,289 (71.7)
	1	39,615 (21.2)
	2	10,568 (5.7)
	3 +	2714 (1.5)
Miscarriage	Yes	34,719 (18.6)
Major congenital anomalies	Yes	5745 (3.1)

^a^Denominator = 181,499 as ethnicity was missing for 5687 women

^b^Denominator = 186,987 as Townsend score was missing for 199 women

^c^Denominator = 102,038, only available where variable could be derived in HES

^d^Denominator = 139,661, as women who only experienced a miscarriage, major congenital anomaly, or termination were not included in either the healthy pregnancy or the APO category

### Description of repeat cardiometabolic risk factor measurements

BP was the most frequently recorded cardiometabolic risk factor, with 94% of women having at least one measurement (Table 2). Women with at least one BP measurement were on average older at their first pregnancy (mean age 27.2 vs. 24.9 years), more likely to be of White ethnicity (86.9% vs. 79.6%), and in the least deprived Townsend score quintile (21.5% vs. 15.8%) (Table 3). The median number of BP measurements per woman was 6 (IQR 3, 12, range 1–369; Additional file 1: Table S3), and the median age when BP was measured was 27 years (IQR 22, 33; Additional file 1: Table S4). BMI was recorded in 87% of women; these women were on average older at their first pregnancy (mean age 27.5 vs. 24.2 years), slightly more likely to be of White ethnicity (86.6% vs. 85.4%), more likely to be in the least deprived Townsend score quintile (21.7% vs. 17.9%; Table 3), and the median number of measurements per woman was 3 (IQR 2, 6, range 1–174; Additional file 1: Table S3). Cholesterol was recorded in 22% of women; these women were less likely to be of White ethnicity than women without a cholesterol measurement (84.8% vs. 87.0%; Table 3). The median number of cholesterol measurements was 1 (IQR 1, 2, range 1–65), with more measurements taking place after first pregnancy than before (Additional file 1: Table S3). Glucose was recorded in 46% of women; these women were slightly more likely to be of White ethnicity (87.0% vs. 86.1%) and in the least deprived Townsend score quintile (22.1% vs. 20.4%; Table 3). The median number of glucose measurements was 1 (IQR 1, 3, range 1–64), with more measurements occurring after first pregnancy than before (Additional file 1: Table S3). Women with multiple miscarriages were more likely to have at least one measurement of BP (97.2%), BMI (93.1%), and cholesterol (35.5%) compared to the rest of the cohort of women, and women with GD were more likely to have glucose measured (61.4%) (Table 2). Women who only experienced healthy pregnancies were less likely to have cardiometabolic risk factor measurements (BP 91.1%, BMI 85.2%, cholesterol 20.8%, glucose 44.5%; Table 2), but for those with at least one measure, the average number of measurements did not vary much for women with and without APOs (Additional file 1: Table S3).

**Table 2:** Number of women with at least one cardiometabolic risk factor measurement, by APOs

	*N* (%) with at least one measure			
	BP	BMI	Cholesterol	Glucose
All women	176,311 (94.2)	163,590 (87.4)	42,013 (22.4)	89,039 (47.6)
Women with only healthy pregnancies	76,080 (91.1)	71,081 (85.2)	17,365 (20.8)	37,117 (44.5)
Women with gestational hypertension	16,949 (95.9)	15,917 (90.0)	5468 (30.9)	9587 (54.2)
Women with PE	7197 (95.8)	6738 (89.7)	2252 (30.0)	4089 (54.4)
Women with GD	7061 (94.8)	6739 (90.5)	2443 (32.8)	4571 (61.4)
Women with spontaneous onset PTB	5494 (95.7)	5104 (88.9)	1446 (25.2)	2997 (52.2)
Women with medically indicated PTB	3040 (95.7)	2858 (89.9)	1020 (32.1)	1882 (59.2)
Women with SGA	11,976 (94.7)	11,237 (88.9)	2912 (23.0)	6446 (51.0)
Women with LGA	11,530 (96.3)	10,879 (90.8)	3233 (27.0)	6528 (54.5)
Women with 3 + miscarriages	1086 (97.2)	1040 (93.1)	397 (35.5)	676 (60.5)
Women with stillbirth	1225 (94.4)	1148 (88.5)	333 (25.7)	654 (50.4)
Women with miscarriage	33,073 (95.3)	31,020 (89.3)	9151 (26.4)	18,044 (52.0)
Women who experienced pregnancy with major congenital anomalies	5567 (96.9)	5291 (92.1)	1645 (28.6)	3206 (55.8)

**Table 3:** Characteristics of women by 1 + vs. no cardiometabolic risk factor

	BP		BMI		Cholesterol		Glucose	
	None	At least one	None	At least one	None	At least one	None	At least one
Age at first pregnancy, mean (SD)								
	24.9 (7.1)	27.2 (6.6)	24.2 (6.8)	27.5 (6.5)	26.2 (6.4)	30.2 (6.5)	26.5 (6.5)	27.8 (6.7)
Ethnicity, *N* (%)^a^								
White	8127 (79.6)	148,841 (86.9)	19,223 (85.4)	137,745 (86.6)	122,141 (87.0)	34,827 (84.8)	83,105 (86.0)	73,863 (87.0)
Non-White	1610 (15.8)	16,894 (9.9)	2385 (10.6)	16,119 (10.1)	13,389 (9.5)	5115 (12.5)	9557 (9.9)	8947 (10.5)
Unknown	478 (4.7)	5549 (3.2)	904 (4.0)	5123 (3.2)	4896 (3.5)	1131 (2.8)	3926 (4.1)	2101 (2.5)
Patient level Townsend score quintile, *N* (%)^b^								
1 (least deprived)	1713 (15.8)	37,928 (21.5)	4213 (17.9)	35,428 (21.7)	29,690 (20.5)	9951 (23.7)	20,445 (20.4)	19,196 (22.1)
2	1953 (18.0)	36,102 (20.5)	4313 (18.3)	33,742 (20.7)	29,217 (20.2)	8838 (21.1)	20,165 (20.1)	17,890 (20.6)
3	2163 (19.9)	36,923 (21.0)	4763 (20.2)	34,323 (21.0)	30,574 (21.1)	8512 (20.3)	21,030 (21.0)	18,056 (20.8)
4	2633 (24.2)	37,724 (21.4)	5497 (23.3)	34,860 (21.3)	31,895 (22.0)	8462 (20.2)	22,006 (22.0)	18,351 (21.2)
5 (most deprived)	2401 (22.1)	27,447 (15.6)	4776 (20.3)	25,072 (15.3)	23,626 (16.3)	6222 (14.8)	16,593 (16.6)	13,255 (15.3)

^a^Ethnicity was missing for 5824 women

^b^Townsend score was missing for 199 women

### Cardiometabolic risk factor trajectories

**Fig. 2:**
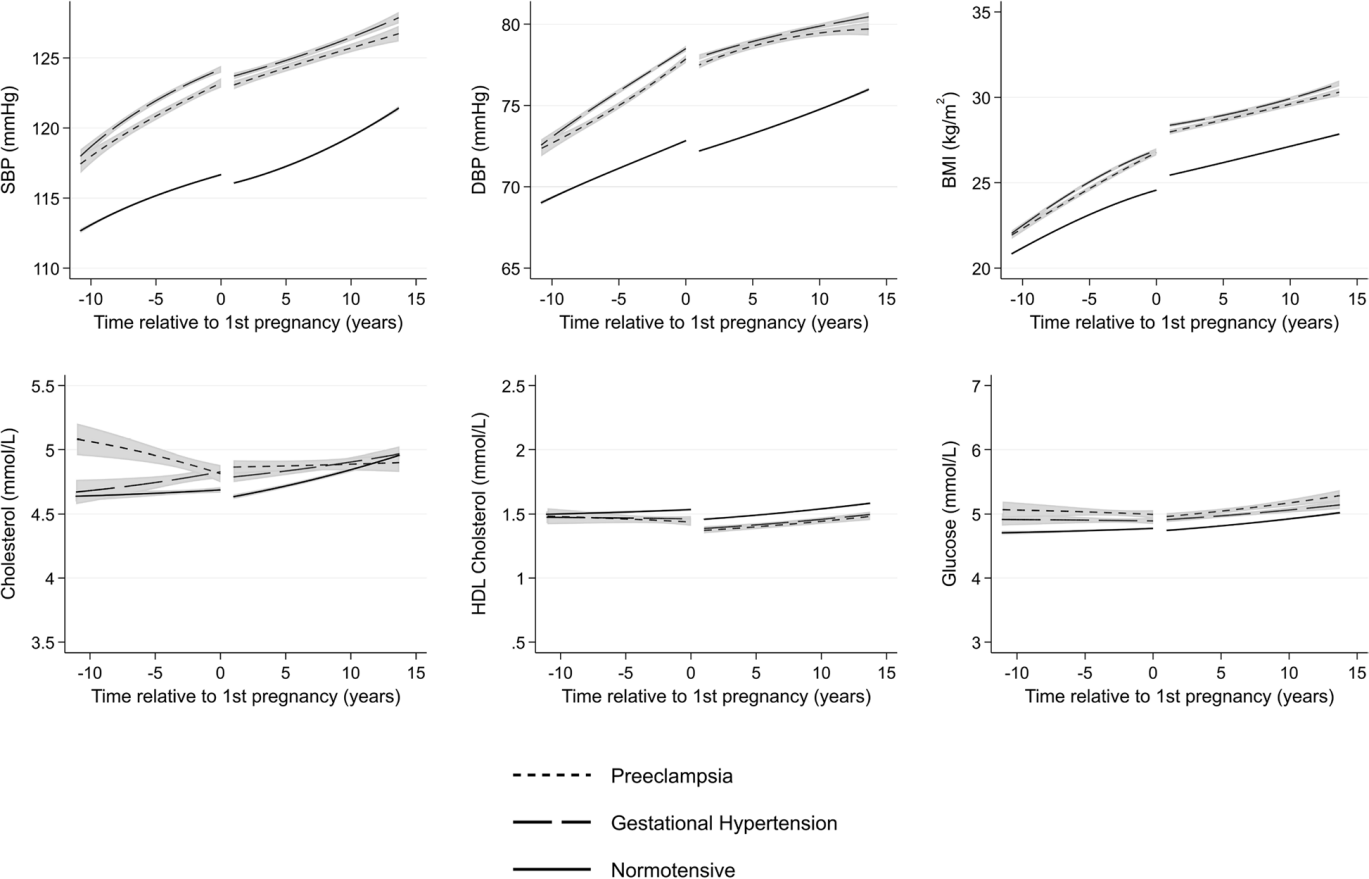
Trajectories of cardiometabolic risk factors by hypertensive disorders of pregnancy

Levels of all cardiovascular risk factors increased with age (Additional file 1: Figs. S1–S11). Women who had a recording of PE or gestational hypertension in at least one pregnancy already had higher mean BMI, BP, and glucose 10 years before first pregnancy compared with women with normotensive pregnancies (Fig. 2). Higher pre-pregnancy levels of both systolic and diastolic BP were observed among women with PE and gestational hypertension compared to women with normotensive pregnancies, and differences continued to 15 years post-pregnancy. Women with PE had higher total cholesterol compared to women with normotensive pregnancies 10 years before their first pregnancy; however, by 10 years after first pregnancy, mean levels were similar. Mean levels of HDL cholesterol were overlapping 10 years before first pregnancy but were slightly lower for women with PE and gestational hypertension after first pregnancy compared to women with normotensive pregnancies and remained lower to 15 years post-pregnancy.

**Fig. 3:**
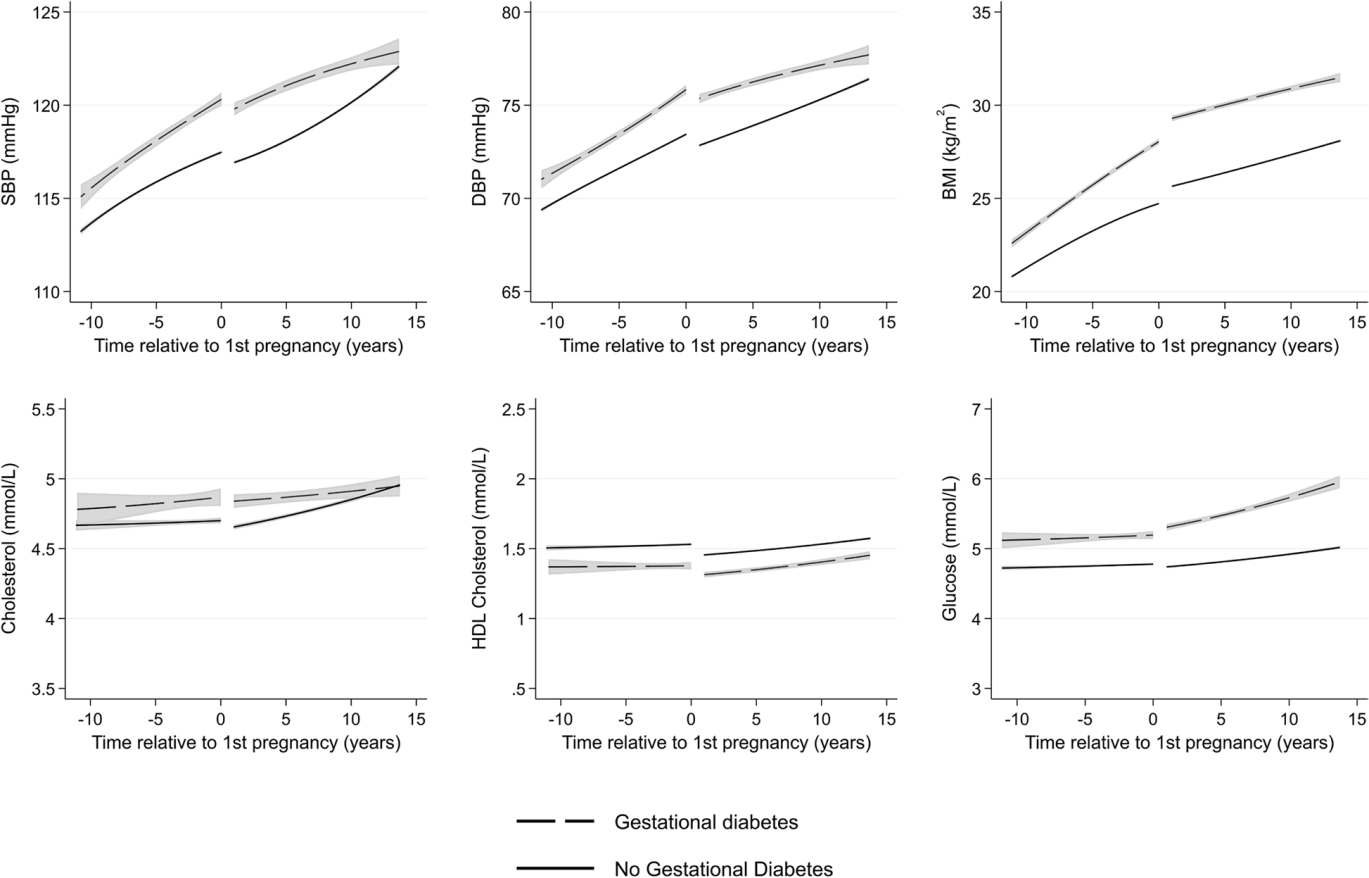
Trajectories of cardiometabolic risk factors by gestational diabetes

Women with GD had higher BMI, BP, and glucose across the life course, with trajectories broadly parallel, though a steeper post-partum rise in glucose was observed in women with vs. without GD (Fig. 3). They also had slightly higher total cholesterol and slightly lower HDL cholesterol before and after first pregnancy, compared to women without GD.

**Fig. 4:**
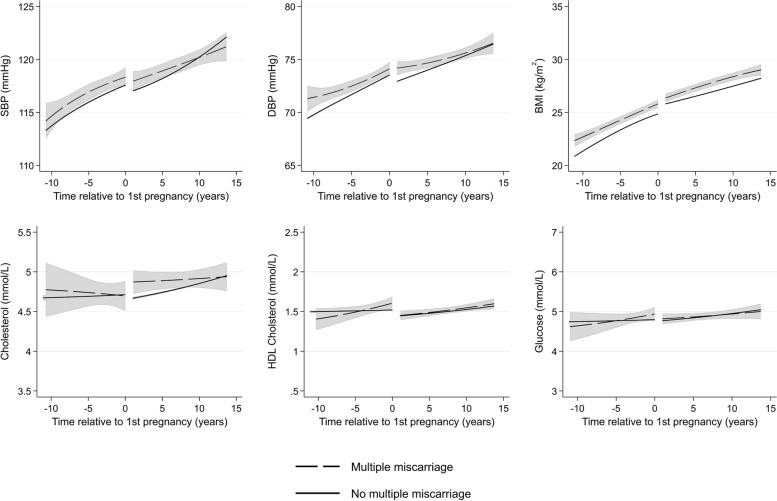
Trajectories of cardiometabolic risk factors by multiple miscarriages

**Fig. 5:**
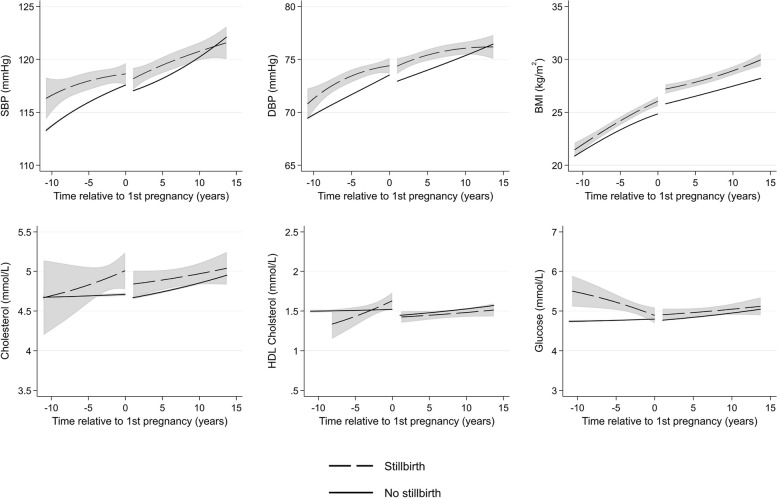
Trajectories of cardiometabolic risk factors by stillbirth

Women with multiple miscarriages (Fig. 4) and stillbirth (Fig. 5) had higher mean BP before their first pregnancy compared to women who did not experience these events, but with wide CIs, and mean levels were similar by 15 years after pregnancy. They also had higher BMI 10 years before their first pregnancy, and the difference continued for 15 years after their first pregnancy, compared to women without these conditions. Women with multiple miscarriages had similar total cholesterol before pregnancy but higher total cholesterol shortly after first pregnancy compared to women without multiple miscarriages. However, by 15 years after pregnancy, mean levels were similar. Women with stillbirth had higher total cholesterol 5 years before and 5 years after first pregnancy, but CIs were wide, and trajectories were similar to women without stillbirth at 10 years before and 10 years after first pregnancy. Little difference between women with and without multiple miscarriages and stillbirth were observed for HDL cholesterol.

**Fig. 6:**
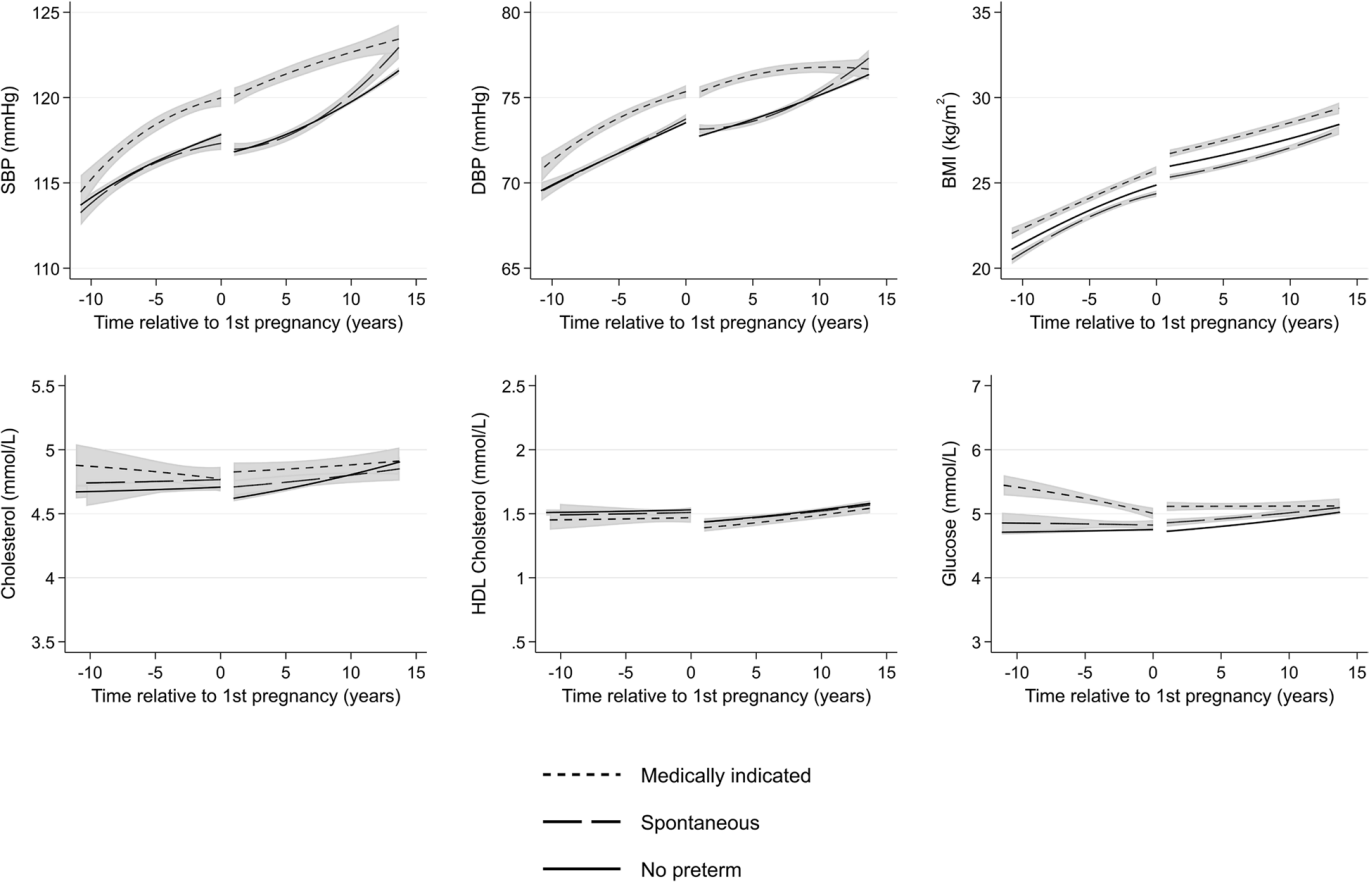
Trajectories of cardiometabolic risk factors by preterm delivery

Women with medically indicated PTB had higher BP, BMI, and glucose before and after pregnancy compared to women with spontaneous PTB and women who did not experience PTB (Fig. 6), with trajectories starting to converge at 15 years after first pregnancy. Women with spontaneous PTB had a lower BMI 10 years before and 15 years after pregnancy compared to women who did not experience PTB. Ten years prior to first pregnancy, total cholesterol was highest in women with medically indicated PTB, second highest in women with spontaneous PTB, and lowest in women without these conditions. However, CIs were wide and mean levels were similar at around 5 years after pregnancy.

**Fig. 7:**
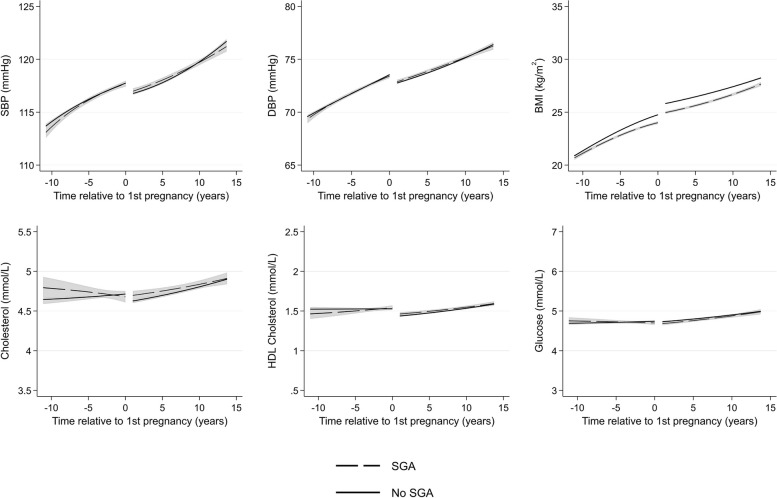
Trajectories of cardiometabolic risk factors by small for gestational age

**Fig. 8:**
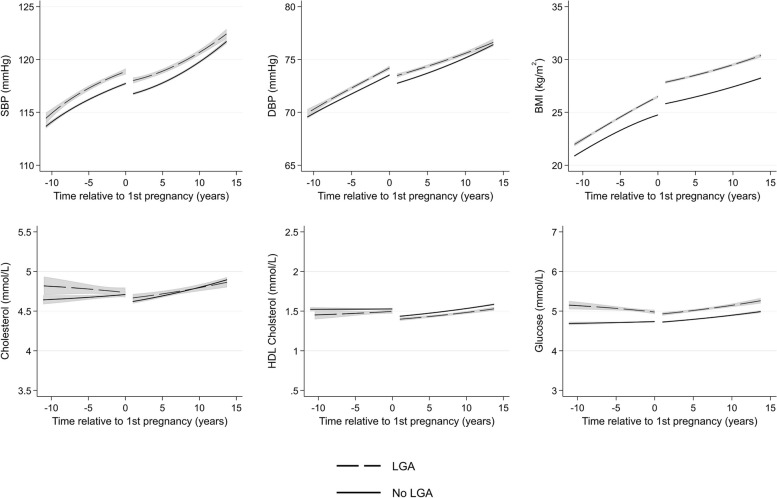
Trajectories of cardiometabolic risk factors by large for gestational age

Women with SGA deliveries have similar BP, HDL cholesterol, and glucose trajectories to women without SGA deliveries (Fig. 7). Mean levels of BMI were similar at 10 years prior to first pregnancy, but women with SGA deliveries had lower BMI from around 5 years prior to first pregnancy, until 15 years post-pregnancy. On the other hand, women who experienced LGA deliveries had higher systolic BP, BMI, and glucose from 10 years before through to 15 years after first pregnancy (Fig. 8). Total cholesterol was slightly higher 10 years prior to pregnancy for women with SGA and LGA, but the differences did not persist post-pregnancy.

**Fig. 9:**
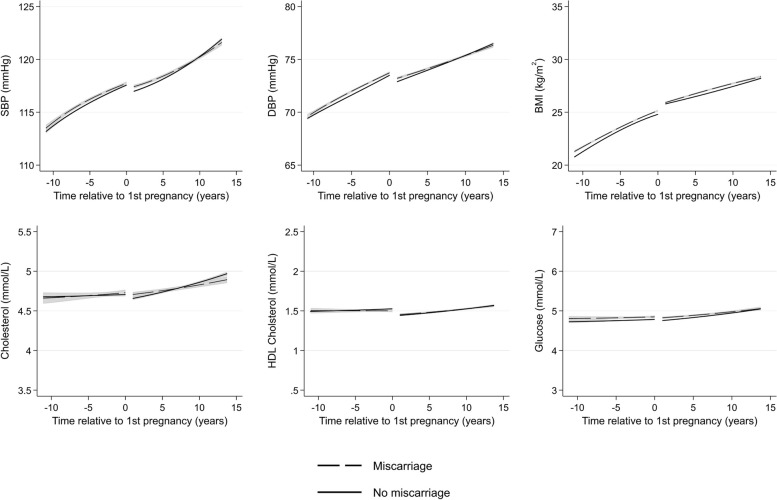
Trajectories of cardiometabolic risk factors by miscarriage

**Fig. 10:**
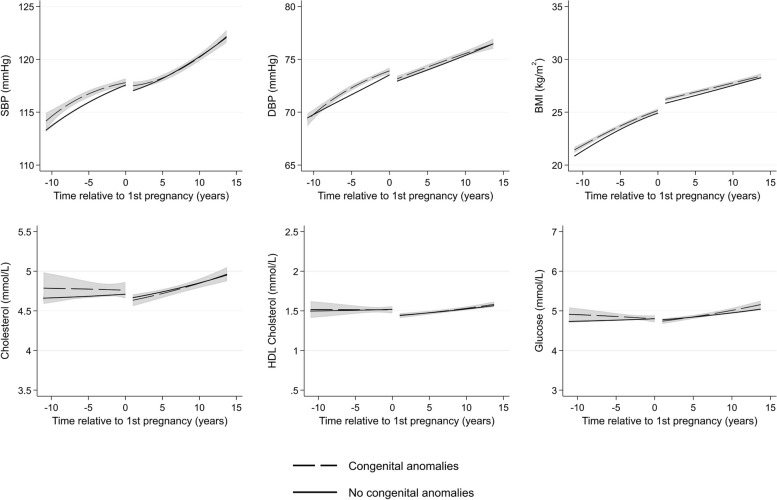
Trajectories of cardiometabolic risk factors by major congenital anomalies

**Fig. 11:**
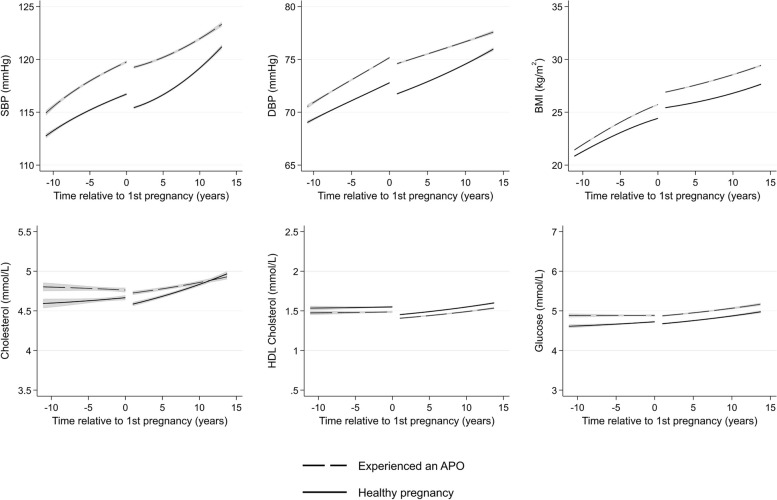
Trajectories of cardiometabolic risk factors by healthy pregnancy versus any adverse pregnancy outcome

**Fig. 12:**
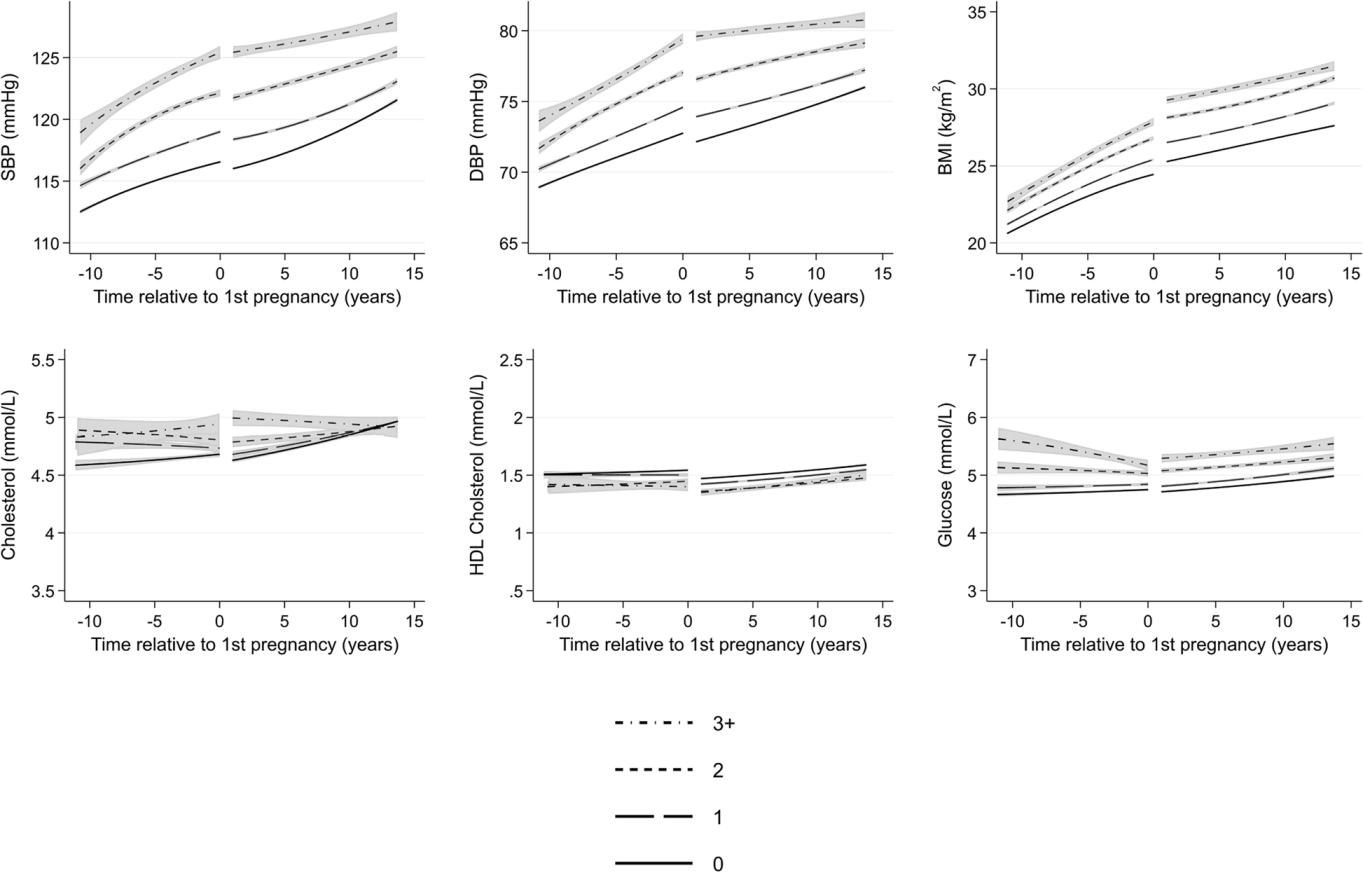
Trajectories of cardiometabolic risk factors by number of adverse pregnancy outcomes

There were few differences in cardiometabolic trajectories between women with and without a history of any miscarriage (Fig. 9) or with offspring with major congenital anomalies (Fig. 10). Women who experienced only healthy pregnancies had favourable levels of cardiometabolic risk factors across all ages compared to women who experienced at least one APO (Fig. 11). Finally, we compared women by the number of APOs. We found a dose–response association between the number of APOs experienced and cardiometabolic risk factors (Fig. 12). This was observed from 10 years before their first pregnancy to 15 years after pregnancy for all cardiometabolic risk factors except for cholesterol, where the mean levels converged in the later years. Additional file 1: Table S5 shows the predicted cardiometabolic risk factors by number of APOs and time relative to first pregnancy and age. For example, predicted systolic BP at 10 years before first pregnancy was 112.9 mmHg (95% CI 112.8, 113.1) for women who experienced no APOs, and was 115.1 (114.8, 115.3), 116.8 (116.3, 117.2), and 119.6 mmHg (118.8, 120.4) for women who experienced 1, 2, and 3 + APOs, respectively. Ten years after first pregnancy, predicted systolic BP was 119.5 (95% CI 119.4, 119.6), 121.2 (121.2, 121.4), 124.3 (124.1, 124.6), and 127.1 mmHg (126.6, 127.5) for women who experienced 0, 1, 2, and 3 + APOs, respectively.

### Sensitivity analyses

Results were broadly similar in sensitivity analysis for women with at least one measure before and one after pregnancy (Additional file 1: Figs. S12–S22), and sensitivity analysis using random intercepts and random slopes for the systolic BP outcome (Additional file 1: Fig. S23a and S23b).

## Discussion

In this large population-based cohort study, women who experienced gestational hypertension, PE, GD, multiple miscarriages, stillbirth, medically indicated PTB, and delivered an LGA baby had adverse levels of modifiable cardiovascular risk factors from 10 years before first pregnancy to up to 15 years after first pregnancy. These findings suggest that women who develop these APOs already have more adverse levels of measured cardiovascular risk factors prior to conception. There were very little differences in any of the cardiometabolic trajectories between women with and without a history of miscarriage or congenital anomalies. We also found that the number of APOs experienced by women over their pregnancy history was associated with more adverse mean levels of cardiometabolic risk factors in a dose–response relationship. Differences were stable pre- and post-pregnancy, except for cholesterol where differences disappeared at 10 years after pregnancy, despite accounting for medication.

Our results are largely in line with what was observed in a previous publication using data from the Norwegian HUNT cohort [8], though our study is considerably larger in scale. In both HUNT and the current study, it was observed that multiple cardiovascular risk factors were already elevated before the first pregnancy in women who later experienced hypertensive disorders of pregnancy compared with women with normotensive first pregnancies. In HUNT, from 40 to 60 years, women with a normotensive pregnancy had a seemingly steeper rise in non-HDL cholesterol, resulting in women with and without PE having similar non-HDL cholesterol levels by age 60 years. In CPRD, we saw a similar pattern with total cholesterol. Consistent with our findings, the HUNT study also found that women with LGA offspring had more adiposity and less favourable glucose measures from prior to first birth and into later life [9]. However, in contrast to HUNT, we observed less favourable BP trajectories for women who experienced LGA, whereas in HUNT less favourable BP trajectories were observed for women who experienced SGA but not LGA. There was little evidence of differences in cardiovascular risk factors between women with normotensive preterm birth compared to women who delivered at term [10]. Similarly, we observed that women with medically indicated PTB had higher BP, BMI, and glucose before and after pregnancy compared to women who did not experience PTB, but not women with spontaneous PTB. These findings are consistent with the link between induced PTB and PE.

We extended existing evidence and examined long-term trajectories of cardiovascular risk factors among women with a history of miscarriage, multiple miscarriages, stillbirth, offspring with major congenital anomalies, and the number of APOs experienced. Our finding that cardiometabolic risk profiles are more adverse in women with multiple APOs are in line with a recent report based on a large national Swedish cohort, which found that women who experienced any of PTB, delivering a SGA baby, PE, other hypertensive disorders of pregnancy, or GD showed an increased risk for ischemic heart disease up to 46 years after delivery, and women who experienced multiple APOs showed further increases in risk [2]. For example, for up to 10 years after delivery, hazard ratios for ischemic heart disease after experiencing 1, 2, or ≥ 3 APOs were 1.29 (95% CI 1.19 to 1.39), 1.80 (1.59 to 2.03), and 2.26 (1.89 to 2.70), respectively, compared with women who never experienced an APO, after adjusting for maternal sociodemographic factors, parity, and traditional cardiovascular risk factors. Our findings are also in line with results from a large Norwegian population study, which found that pregnancy histories with complications at 40 years of age were strongly associated with subsequent risk of premature death from atherosclerotic CVD; this risk increased with increasing number of complicated pregnancies [14].

Our study adds to the growing body of evidence suggesting that maternal cardiometabolic health before pregnancy is associated with APO risk; several studies have demonstrated that pre-pregnancy and first-trimester cardiometabolic factors are associated with APO risk [25–28]. Furthermore, a recent study performed a sex-stratified phenome-wide association analysis of polygenic risk scores for PE and gestational hypertension [29]. The risk scores were strongly associated with phenotypes such as hypertension, type II diabetes, and ischemic heart disease in both sexes. The similar associations between sexes suggest that most genes identified are not pregnancy-specific but rather that pregnancy likely unmasks underlying cardiovascular risk. Additionally, a recent study from the Norwegian Mother, Father and Child Cohort and the Trøndelag Health Study further support this by showing that women—but not men—with a genetically predicted liability for coronary heart disease (CHD) had an increased risk of any hypertensive disorders of pregnancy, PE, and delivering SGA infants [30].

Our study’s strengths include using CPRD GOLD, which provides a large longitudinal dataset, comprehensive health data, repeat measures of cardiometabolic risk factors within women, and a validated source of pregnancy information. However, there are also limitations to our study. Women with measures of cardiometabolic traits before their first pregnancy are different to those who do not have these measures. They are likely to be older, have symptoms that have led to the measures being taken, or might be using hormonal contraception, necessitating blood pressure monitoring. Additionally, they might be taking other medications that would indicate monitoring of these factors. These indications for having cardiometabolic traits measured in turn potentially influence APOs. Therefore, availability of cardiometabolic data is subject to ‘information presence and observation’ [31]. This implies that availability of measures of cardiometabolic traits might follow the same principle as informative censoring or non-ignorable missing data. Women who experienced APOs were more likely to have cardiometabolic risk factors measured than those who did not experience APOs. This could introduce bias if there is an interaction between the unobserved cardiometabolic measures and the occurrence of APOs in determining the likelihood of these risk factors being measured. This interaction seems unlikely before pregnancy. Women who had an APO may be followed up more frequently after pregnancy, but it seems unlikely that this is influenced by the true value of the unobserved measures. Moreover, selection bias cannot be ruled out as overall healthy women are likely to be under-represented in the analysis of cholesterol and glucose, therefore, potentially biasing associations. We controlled for smoking status, ethnicity, and deprivation in analyses, but these factors alone are unlikely to account for all the reasons cardiometabolic tests were conducted. We also had to exclude women if all pregnancy outcomes were unknown; some of these pregnancies were ongoing during data extraction, but other unknown pregnancy outcomes may include undocumented miscarriages or terminations. We could only identify major congenital anomalies from offspring records and, therefore, did not have this information in women who had terminations. CIs were wide for some of the less frequently measured cardiometabolic outcomes, particularly at the younger ages where there were less data. Finally, we may have misclassified APO status; we did not have a complete pregnancy history on all women, as some women were young at the end of follow-up and may have further pregnancies after the period covered by our data capture. As with any study addressing miscarriage, we have likely missed miscarriages not registered as such.

## Conclusions

Women who experienced APOs had adverse cardiometabolic health profiles prior to first pregnancy, and differences persisted up to 15 years post-pregnancy. Our findings highlight the potential for targeted public health interventions aimed at promoting good cardiometabolic health in young adults as they transition from contraceptive use to planning pregnancies. APOs may identify young women who could benefit from monitoring of CVD risk factors and interventions to improve cardiometabolic health in the post-partum years.

## Supplementary Information

Additional file 1: Supplementary information, tables, and figures. Text S1 Further details on APO coding. Text S2 Further information on fractional polynomials and multilevel models. Table S1 Median registration time at GP practices, before and after first pregnancy. Table S2 Sample comparison table. Table S3 Number of cardiometabolic risk factor measurements, for women with at least one measure. Table S4 Median age at cardiometabolic risk factor measurement, for women with at least one measure. Table S5 Predicted mean levels of cardiometabolic risk factors by number of APOs, time relative to first pregnancy, and age. Figures S1–S11 Trajectories of cardiometabolic risk factors by APOs, with age on the x-axis. Figures S12–S22 Trajectories of cardiometabolic risk factors by APOs, for women with a cardiometabolic measurement before and after pregnancy. Figure S23a and S23b Sensitivity analysis using random intercepts and random slopes for the systolic BP outcome, trajectories by APO

## Data Availability

This study is based on data from CPRD obtained under licence from the UK Medicines and Healthcare products Regulatory Agency. The data is provided by patients and collected by the NHS as part of their care and support. The interpretation and conclusions contained in this study are those of the authors alone. Access to CPRD data is subject to approval by the CPRD Research Data Governance (RDG) process (https://cprd.com/data-access). Our CPRD protocol was approved by the Independent Scientific Advisory Committee (ISAC; protocol number: 20_145R); approval via ISAC has now been replaced by the RDG process.

## Abbreviations

APO: Adverse pregnancy outcome
BMI: Body mass index
BP: Blood pressure
CI: Confidence interval
CPRD: Clinical Practice Research Datalink
CVD: Cardiovascular disease
DBP: Diastolic BP
EUROCAT: European Surveillance of Congenital Anomalies
GD: Gestational diabetes
HDL: High-density lipoprotein
HES: Hospital Episode Statistics
IQR: Interquartile range
LGA: Large for gestational age
PE: Preeclampsia
PTB: Preterm birth
SBP: Systolic BP
SGA: Small for gestational age
SD: Standard deviation
UTS: Up to standard
