# Supplementary material for: Life course trajectories of maternal cardiovascular disease risk factors by obstetric history: a UK cohort study using electronic health records
Source: BMC Med. 2025 Feb 14;23:91. doi: 10.1186/s12916-025-03937-y (PMC11827161; doi:10.1186/s12916-025-03937-y)
Supplement: Supplementary file 1 — Additional file 1: Supplementary information, tables, and figures. Text S1 Further details on APO coding. Text S2 Further information on fractional polynomials and multilevel models. Table S1 Median registration time at GP practices, before and after first pregnancy. Table S2 Sample comparison table. Table S3 Number of cardiometabolic risk factor measurements, for women with at least one measure. Table S4 Median age at cardiometabolic risk factor measurement, for women with at least one measure. Table S5 Predicted mean levels of cardiometabolic risk factors by number of APOs, time relative to first pregnancy, and age. Figures S1–S11 Trajectories of cardiometabolic risk factors by APOs, with age on the x-axis. Figures S12–S22 Trajectories of cardiometabolic risk factors by APOs, for women with a cardiometabolic measurement before and after pregnancy. Figure S23a and S23b Sensitivity analysis using random intercepts and random slopes for the systolic BP outcome, trajectories by APO [file 12916_2025_3937_MOESM1_ESM.docx]

# Life course trajectories of maternal cardiovascular disease risk factors by obstetric history: a UK cohort study using electronic health records

# Additional file 1: Supplementary information, tables, and figures

**Text S1 Further details on adverse pregnancy outcome (APO) coding**

APOs of interest were gestational hypertension, preeclampsia (PE), gestational diabetes (GD), preterm birth (PTB), small or large for gestational age (SGA, LGA), miscarriage, multiple miscarriages, stillbirth, and offspring with major congenital anomalies. For the creation of a combined three-level hypertensive disorders of pregnancy variable, if a women had ever experienced PE and gestational hypertension in pregnancies, they were coded in the PE group. For the creation of the three-level PTB variable, if a woman had ever experienced spontaneous onset and medically indicated preterm deliveries in pregnancy, they were coded in the spontaneous onset PTB group.

**Text S2 Further information on fractional polynomials and multilevel models**

Multilevel models with random intercepts were used to estimate trajectories of each cardiometabolic risk factor with differing time axes: (1) over time relative to first pregnancy, and (2) over age, for women with and without each APO.

Fractional polynomial models were used to model non-linear relationships of cardiometabolic risk factors over time/age, to generate smooth curves of predicted mean levels. To fit the fractional polynomials the x-variables were first scaled to avoid extreme values being created from the power transformations. Age was scaled as: [scaled age = age/10]; time relative to first pregnancy was scaled as: [time scaled = (time/10)+4]. Model fit was assessed using the change in deviance.

The best fitting models with up to 2 degrees were chosen with the following powers:

Systolic BP: -2, 3 (age); -2, 3 (time)

Diastolic BP: -2, 3 (age); -2, 3 (time)

BMI: 3 (age); 3 (time)

Total cholesterol: 2 (age); 3 (time)

HDL cholesterol: 3 (age); 3 (time)

Glucose: -2, 2 (age); -2, 3 (time)

Including an interaction between each of the polynomial terms and the APO allows us to examine whether average cardiometabolic trajectories vary between women with and without an APO. In the analysis with time on the x-axis, we further increased the flexibility of the trajectories by additionally including an interaction term for whether the cardiometabolic measure was before or after the first pregnancy. Models controlled for age at first pregnancy, Townsend residential area deprivation score quintile, whether the women had experienced non-singleton pregnancies, the total number of pregnancies for each woman, smoking status, ethnicity, and use of medicines. Analyses were performed in Stata 18. We obtained estimates of the population mean levels and standard errors using Stata’s postestimation commands, at average levels of the included covariates: 27 years at first pregnancy, non-singleton pregnancies, a total of 2 pregnancies, Townsend residential area deprivation score quintile 3, White ethnicity, no use of medicines. We plotted the predicted mean levels of cardiometabolic risk factors and 95% confidence intervals over age or time for women with and without each APO. We excluded cardiometabolic measurements taken during pregnancy and thus added a gap in the risk factor trajectories in the analysis with time on the x-axis.

**Table S1 Median (interquartile range) of registration time (years) at GP practices, before and after first pregnancy**

| Year of first pregnancy |  | Registration time (years) before first pregnancy | | Data collection time (years) after first pregnancy | |
| --- | --- | --- | --- | --- | --- |
|  | N | Median | IQR | Median | IQR |
| 1997 | 5,036 | 5.1 | (1.9, 13.1) | 17.1 | (15.1, 18.7) |
| 1998 | 5,366 | 5.0 | (1.8, 13.7) | 16.2 | (14.3, 17.8) |
| 1999 | 6,205 | 5.1 | (1.8, 13.4) | 15.3 | (13.4, 17.2) |
| 2000 | 7,854 | 4.9 | (1.7, 12.7) | 14.4 | (12.5, 16.4) |
| 2001 | 8,871 | 4.9 | (1.7, 13.1) | 13.5 | (11.8, 15.3) |
| 2002 | 10,163 | 4.8 | (1.8, 13.2) | 12.5 | (10.7, 14.8) |
| 2003 | 11,117 | 4.8 | (1.7, 13.2) | 11.5 | (9.7, 13.8) |
| 2004 | 11,531 | 5.1 | (1.8, 14.0) | 10.5 | (8.7, 12.9) |
| 2005 | 11,784 | 5.2 | (1.8, 14.8) | 9.5 | (7.7, 11.8) |
| 2006 | 11,924 | 5.1 | (1.8, 14.7) | 8.5 | (6.6, 11.0) |
| 2007 | 12,396 | 5.1 | (1.7, 15.2) | 7.5 | (5.7, 9.9) |
| 2008 | 12,413 | 5.3 | (1.8, 15.7) | 6.5 | (4.8, 8.9) |
| 2009 | 12,133 | 5.2 | (1.9, 16.1) | 5.6 | (4.0, 8.1) |
| 2010 | 11,474 | 5.3 | (1.9, 16.3) | 4.8 | (3.3, 7.2) |
| 2011 | 10,952 | 5.3 | (1.9, 16.2) | 3.9 | (2.5, 6.4) |
| 2012 | 9,830 | 5.4 | (1.8, 16.5) | 3.0 | (1.8, 5.8) |
| 2013 | 8,335 | 5.1 | (1.8, 16.3) | 2.5 | (1.3, 5.5) |
| 2014 | 6,459 | 5.1 | (1.7, 15.8) | 2.3 | (0.9, 5.0) |
| 2015 | 4,533 | 5.0 | (1.6, 16.8) | 3.1 | (1.1, 4.3) |
| 2016 | 3,269 | 4.4 | (1.5, 14.3) | 3.0 | (1.5, 3.5) |
| 2017 | 2,648 | 4.0 | (1.5, 14.5) | 2.2 | (1.4, 2.6) |
| 2018 | 1,991 | 4.2 | (1.6, 15.5) | 1.3 | (1.0, 1.7) |
| 2019 | 902 | 4.5 | (1.7, 16.0) | 0.7 | (0.6, 0.9) |
| Overall | 187,186 | 5.1 | (1.8, 14.9) | 7.9 | (4.1, 12.2) |

Notes: Time before first pregnancy is CPRD GOLD current registration date to pregnancy start date, in years. Time after first pregnancy is first pregnancy end date to last collection date or date of death, in years.

**Table S2 Sample comparison table**

|  | Women with a pregnancy in the pregnancy register, starting 1 Jan 1997 - 31 Dec 2019 | Restricting to women with first pregnancies in the Pregnancy Register | Restrict to UTS practices | Registered at UTS practice at least 3m before pregnancy start | Eligible for linkage^4^ | Study population (excluding women with all unknown pregnancy outcomes) |
| --- | --- | --- | --- | --- | --- | --- |
| N | 1,742,073 | 1,403,839 | 1,043,409 | 475,143 | 228,297 | 187,186 |
| Age 1st preg | N/A^1^ | 26.1 (6.5)^2^ | 26.1 (6.5)^3^ | 27.0 (6.6) | 27.1 (6.7) | 27.1 (6.6) |
| Experienced multiple preg | N/A^1^ | 11,954 (0.9) | 8,826 (0.9) | 4,207 (0.9) | 2,178 (1.0) | 2,020 (1.1) |
| Parity |  |  |  |  |  |  |
| 1 | N/A^1^ | 586,233 (41.8) | 450,569 (43.2) | 226,259 (47.6) | 107,734 (47.2) | 77,951 (41.6) |
| 2 | N/A^1^ | 381,349 (27.2) | 286,031 (27.4) | 125,726 (26.5) | 60,704 (26.6) | 52,643 (28.1) |
| 3 | N/A^1^ | 214,732 (15.3) | 156,107 (15.0) | 63,881 (13.4) | 30,717 (13.5) | 28,116 (15.0) |
| 4 | N/A^1^ | 112,832 (8.0) | 79,367 (7.6) | 31,595 (6.7) | 15,518 (6.8) | 14,994 (8.0) |
| 5+ | N/A^1^ | 108,693 (7.7) | 71,335 (6.8) | 27,682 (5.8) | 13,624 (6.0) | 13,482 (7.2) |
| Ethnicity |  |  |  |  |  |  |
| White | 642,609 (81.2) | 522,980 (80.0) | 399,666 (81.4) | 186,902 (85.8) | 186,902 (85.8) | 156,968 (86.5) |
| Non-white | 118,873 (15.0) | 106,821 (16.3) | 74,785 (15.2) | 23,711 (10.9) | 23,711 (10.9) | 18,504 (10.2) |
| Unknown | 30,232 (3.8) | 23,888 (3.7) | 16,454 (3.4) | 7,342 (3.4) | 7,342 (3.4) | 6,027 (3.3) |
| Missing | 950,359 | 653,689 | 552,504 | 257,188 | 10,342 | 5,687 |
| Townsend |  |  |  |  |  |  |
| 1 (least deprived) | 156,888 (18.8) | 126,211 (18.3) | 101,213 (19.6) | 46,881 (20.6) | 46,881 (20.6) | 39,641 (21.2) |
| 2 | 156,849 (18.8) | 127,225 (18.45) | 99,392 (19.3) | 45,670 (20.0) | 45,670 (20.0) | 38,055 (20.4) |
| 3 | 172,984 (20.73) | 143,071 (20.74) | 106,814 (20.7) | 47,843 (21.0) | 47,843 (21.0) | 39,086 (20.9) |
| 4 | 188,977 (22.65) | 158,013 (22.91) | 114,867 (22.3) | 50,019 (21.9) | 50,019 (21.9) | 40,357 (21.6) |
| 5 (most deprived) | 158,661 (19.02) | 135,151 (19.6) | 93,155 (18.1) | 37,606 (16.5) | 37,606 (16.5) | 29,848 (16.0) |
| Missing | 907,714 | 714,168 | 515,441 | 247,124 | 278 | 199 |

^1^ N/A because information is not available on complete pregnancy history for all women. ^2^ 25,173 (1.8%) have missing year of birth information. ^3^ 15,400 (1.5%) have missing year of birth information. ^4^ A subset of English practices have consented to participate in the CPRD linkage scheme.

**Table S3** **Number of cardiometabolic risk factors measurements, for women with at least one measure**

|  | **BP** | **BMI** | **Cholesterol** | **Glucose** |
| --- | --- | --- | --- | --- |
| **Pre-first pregnancy** | **Median; Lower quartile, Upper quartile; Range** | | | |
| All women | 3; 1, 6; 0-205 | 2; 1, 3; 0-116 | 0; 0, 1; 0-25 | 1; 0, 1; 0-24 |
| Women with: |  |  |  |  |
| Only healthy pregnancies | 3; 1, 6; 0-176 | 2; 1, 3; 0-116 | 0; 0, 1; 0-24 | 1; 0, 1; 0-24 |
| Gestational hypertension | 3; 1, 7; 0-205 | 2; 1, 3; 0-58 | 0; 0, 1; 0- 25 | 0; 0, 1; 0-20 |
| PE | 3; 1, 7; 0-76 | 2; 1, 4; 0-64 | 0; 0, 1; 0-17 | 1; 0, 1; 0-20 |
| GD | 3; 1, 6; 0-85 | 2; 1, 4; 0-54 | 0; 0, 1; 0-23 | 1; 0, 1; 0-16 |
| Spontaneous onset PTB | 3; 1, 7; 0-85 | 2; 1, 3; 0-65 | 0; 0, 1; 0-18 | 0; 0, 1; 0-12 |
| Medically indicated PTB | 3; 1, 7; 0-71 | 2; 1, 4; 0-34 | 0; 0, 1; 0-20 | 1; 0, 1; 0-20 |
| SGA | 3; 1, 6; 0-78 | 2; 1, 3; 0- 43 | 0; 0, 1; 0-13 | 1; 0, 1; 0-24 |
| LGA | 3; 1, 7; 0-55 | 2; 1, 3; 0-94 | 0; 0, 1; 0-25 | 0; 0, 1; 0-21 |
| 3+ miscarriages | 3; 1, 7; 0-38 | 2; 1, 3; 0-27 | 0; 0, 1; 0-13 | 0; 0, 1; 0-9 |
| Stillbirth | 2; 1, 5; 0-35 | 1; 1, 3; 0-33 | 0; 0, 1; 0- 9 | 0; 0, 1; 0-15 |
| Miscarriage | 3; 1, 6; 0-176 | 2; 1, 3; 0-89 | 0; 0, 1; 0-17 | 0; 0, 1; 0-20 |
| Congenital anomalies offspring | 3; 1, 6; 0-46 | 2; 1, 3; 0-65 | 0; 0, 1; 0-12 | 0; 0, 1; 0-15 |
| **Post-first pregnancy** |  |  |  |  |
| All women | 3; 0, 6; 0- 320 | 2; 0, 4; 0-174 | 1; 0, 2; 0-65 | 1; 0, 2; 0-64 |
| Women with: |  |  |  |  |
| Only healthy pregnancies | 3; 0, 6; 0-320 | 2; 0, 4; 0-174 | 1; 0, 2; 0-26 | 1; 0, 2; 0-39 |
| Gestational hypertension | 4; 2, 8; 0-161 | 2; 0, 4; 0-100 | 1; 1, 2; 0-65 | 1; 0, 3; 0-64 |
| PE | 4; 2, 9; 0-153 | 2; 0, 4; 0-100 | 1; 1, 2; 0-29 | 1; 0, 3; 0-29 |
| GD | 3; 0, 6; 0-153 | 2; 0, 4; 0-78 | 1; 0, 3; 0-36 | 1; 0, 3; 0-24 |
| Spontaneous onset PTB | 4; 2, 7; 0-78 | 2; 0, 4; 0-126 | 1; 1, 2; 0-39 | 1; 0, 2; 0-19 |
| Medically indicated PTB | 4; 2, 8; 0-153 | 2; 0, 5; 0-126 | 1; 1, 2; 0-65 | 1; 0, 3; 0-64 |
| SGA | 3; 1, 7; 0-161 | 2; 0, 4; 0-58 | 1; 0, 2; 0-27 | 1; 0, 2; 0-38 |
| LGA | 4; 2, 7; 0-116 | 2; 0, 5; 0-72 | 1; 1, 2; 0-29 | 1; 0, 2; 0-29 |
| 3+ miscarriages | 4; 2, 8; 0-78 | 2; 0, 4; 0-71 | 1; 1, 2; 0-26 | 2; 1, 3; 0-15 |
| Stillbirth | 3; 2, 7; 0-64 | 2; 0, 5; 0-78 | 1; 1, 2; 0-16 | 1; 1, 3; 0-12 |
| Miscarriage | 3; 0, 6; 0-124 | 2; 0, 4; 0-100 | 1; 0, 2; 0- 65 | 1; 0, 2; 0-64 |
| Congenital anomalies offspring | 4; 2, 8; 0-92 | 2; 0, 5; 0-126 | 1; 1, 2; 0-20 | 1; 1, 3; 0-27 |
| **Total** |  |  |  |  |
| All women | 6; 3, 12; 1-369 | 3; 2, 6; 1-174 | 1; 1, 2; 1-65 | 1; 1, 3; 1-64 |
| Women with: |  |  |  |  |
| Only healthy pregnancies | 6; 3, 11; 1-369 | 3; 2, 6; 1-174 | 1; 1, 2; 1-27 | 1; 1, 2; 1-39 |
| Gestational hypertension | 8; 4, 15; 1-207 | 4; 2, 7; 1-100 | 1; 1, 3; 1-65 | 2; 1, 3; 1-64 |
| PE | 8; 4, 15; 1-169 | 4; 2, 7; 1-100 | 1; 1, 3; 1-30 | 2; 1, 3; 1-29 |
| GD | 6; 3, 12; 1-169 | 4; 2, 7; 1-81 | 1; 1, 3; 1-36 | 2; 1, 3; 1- 24 |
| Spontaneous onset PTB | 7; 3, 13; 1-86 | 4; 2, 7; 1-126 | 1; 1, 2; 1-41 | 2; 1, 3; 1- 22 |
| Medically indicated PTB | 8; 4, 15; 1-169 | 4; 2, 8; 1-126 | 1; 1, 3; 1-65 | 2; 1, 3; 1- 64 |
| SGA | 7; 3, 12; 1-165 | 4; 2, 7; 1-75 | 1; 1, 2; 1-28 | 2; 1, 3; 1-38 |
| LGA | 7; 4, 13; 1-129 | 4; 2, 7; 1-95 | 1; 1, 2; 1-29 | 2; 1, 3; 1-29 |
| 3+ miscarriages | 7; 4, 13; 1-78 | 4; 2, 7; 1-72 | 1; 1, 2; 1-26 | 2; 1, 3; 1- 15 |
| Stillbirth | 6; 3, 12; 1-81 | 4; 2, 7; 1-78 | 1; 1, 3; 1-16 | 2; 1, 3; 1-15 |
| Miscarriage | 6; 3, 12; 1-190 | 3; 2, 6; 1-100 | 1; 1, 2; 1-65 | 2; 1, 3; 1- 64 |
| Congenital anomalies offspring | 8; 4, 13; 1-92 | 4; 2, 7; 1-126 | 1; 1, 2; 1-28 | 2; 1, 3; 1-27 |

**Table S****4 Median (IQR) age at cardiometabolic risk factor measurement (years), for women with at least one measure**

|  | BP | BMI | Cholesterol | Glucose |
| --- | --- | --- | --- | --- |
| All women | 27 (22, 33) | 27 (22, 33) | 36 (29, 42) | 31 (24, 38) |
| Women with only healthy pregnancies | 28 (22, 33) | 28 (22, 33) | 37 (30, 43) | 31 (25, 38) |
| Women with gestational hypertension | 29 (23, 35) | 28 (23, 34) | 35 (29, 41) | 32 (26, 38) |
| Women with PE | 29 (23, 35) | 28 (23, 34) | 35 (29, 41) | 32 (26, 39) |
| Women with GD | 28 (23, 34) | 28 (23, 34) | 35 (29, 41) | 32 (26, 37) |
| Women with spontaneous onset PTB | 27 (22, 33) | 27 (22, 33) | 35 (28, 41) | 31 (25, 37) |
| Women with medically indicated PTB | 28 (23, 35) | 28 (23, 34) | 34 (28, 40) | 31 (25, 37) |
| Women with SGA | 27 (22, 33) | 27 (22, 33) | 35 (29, 41) | 30 (24, 37) |
| Women with LGA | 28 (22, 33) | 28 (23, 33) | 34 (28, 41) | 31 (25, 37) |
| Women with 3+ miscarriages | 30 (24, 37) | 30 (24, 37) | 39 (31, 44) | 34 (28, 41) |
| Women with stillbirth | 28 (22, 35) | 29 (23, 34) | 36 (30, 42) | 31 (25, 38) |
| Women with miscarriage | 28 (23, 35) | 28 (23, 35) | 38 (30, 43) | 32 (26, 39) |
| Women who experienced pregnancy with major congenital anomalies | 28 (23, 34) | 29 (23, 34) | 36 (30, 41) | 32 (26, 38) |

Note: women who had cardiometabolic risk factors measured more than once will contribute multiple age measurements to the calculation of median age.

**Table S5 Predicted mean levels of cardiometabolic risk factors (with 95% confidence intervals) by number of APOs, time relative to first pregnancy, and age**

| APOs | Time^1^ or age | Systolic BP  (mmHg) | Diastolic BP (mmHg) | BMI  (kg/m^2^) | Cholesterol (mmol/L) | HDL Cholesterol (mmol/L) | Glucose (mmol/L) |
| --- | --- | --- | --- | --- | --- | --- | --- |
| 0 | -10 | 112.9 (112.8, 113.1) | 69.2 (69.1, 69.4) | 21.1 (21.0, 21.2) | 4.59 (4.55, 4.63) | 1.51 (1.49, 1.53) | 4.67 (4.64, 4.70) |
| 0 | 0 | 116.6 (116.5, 116.7) | 72.8 (72.7, 72.8) | 24.4 (24.4, 24.5) | 4.68 (4.66, 4.71) | 1.54 (1.53, 1.55) | 4.75 (4.73, 4.77) |
| 0 | 10 | 119.5 (119.4, 119.6) | 74.8 (74.7, 74.9) | 26.9 (26.9, 27.0) | 4.85 (4.83, 4.87) | 1.55 (1.54, 1.56) | 4.89 (4.87, 4.91) |
| 1 | -10 | 115.1 (114.8, 115.3) | 70.6 (70.4, 70.7) | 21.7 (21.6, 21.8) | 4.78 (4.72, 4.85) | 1.50 (1.47, 1.53) | 4.78 (4.73, 4.84) |
| 1 | 0 | 119.0 (118.8, 119.2) | 74.6 (74.5, 74.7) | 25.4 (25.3, 25.5) | 4.73 (4.70, 4.77) | 1.50 (1.49, 1.52) | 4.84 (4.81, 4.87) |
| 1 | 10 | 121.2 (121.1, 121.4) | 76.2 (76.0, 76.3) | 28.2 (28.1, 28.3) | 4.87 (4.84, 4.89) | 1.50 (1.49, 1.51) | 5.01 (4.98, 5.04) |
| 2 | -10 | 116.8 (116.3, 117.2) | 72.2 (71.9, 72.5) | 22.7 (22.5, 22.8) | 4.88 (4.78, 4.98) | 1.40 (1.36, 1.45) | 5.12 (5.03, 5.22) |
| 2 | 0 | 122.1 (121.8, 122.4) | 77.0 (76.8, 77.2) | 26.8 (26.7, 26.9) | 4.80 (4.75, 4.86) | 1.45 (1.42, 1.47) | 5.03 (4.97, 5.08) |
| 2 | 10 | 124.3 (124.1, 124.6) | 78.5 (78.3, 78.7) | 29.7 (29.6, 29.9) | 4.88 (4.83, 4.92) | 1.44 (1.42, 1.45) | 5.23 (5.18, 5.27) |
| 3+ | -10 | 119.6 (118.8, 120.4) | 74.0 (73.4, 74.6) | 23.2 (22.9, 23.6) | 4.84 (4.69, 4.99) | 1.42 (1.34, 1.50) | 5.60 (5.42, 5.77) |
| 3+ | 0 | 125.4 (124.9, 126.0) | 79.4 (79.0, 79.8) | 27.9 (27.6, 28.1) | 4.94 (4.85, 5.03) | 1.40 (1.36, 1.44) | 5.17 (5.07, 5.27) |
| 3+ | 10 | 127.1 (126.6, 127.5) | 80.5 (80.1, 80.8) | 30.8 (30.5, 31.0) | 4.94 (4.88, 5.01) | 1.45 (1.42, 1.48) | 5.46 (5.38, 5.53) |
| 0 | 20y | 114.4 (114.3, 114.5) | 70.5 (70.5, 70.6) | 22.7 (22.7, 22.8) | 4.54 (4.51, 4.56) | 1.48 (1.47, 1.49) | 4.56 (4.54, 4.58) |
| 0 | 30y | 117.1 (117.0, 117.2) | 73.2 (73.1, 73.2) | 25.5 (25.5, 25.6) | 4.66 (4.64, 4.67) | 1.50 (1.50, 1.51) | 4.74 (4.73, 4.75) |
| 0 | 40y | 119.9 (119.8, 120.0) | 75.3 (75.2, 75.3) | 27.4 (27.4, 27.5) | 4.83 (4.81, 4.84) | 1.54 (1.54, 1.55) | 4.89 (4.88, 4.91) |
| 0 | 50y | 123.9 (123.7, 124.1) | 77.9 (77.8, 78.0) | 29.3 (29.3, 29.4) | 5.04 (5.02, 5.07) | 1.61 (1.60, 1.62) | 5.04 (5.00, 5.07) |
| 1 | 20y | 116.5 (116.3, 116.6) | 72.0 (71.9, 72.1) | 23.4 (23.3, 23.4) | 4.66 (4.63, 4.69) | 1.44 (1.43, 1.45) | 4.64 (4.62, 4.67) |
| 1 | 30y | 119.4 (119.3, 119.5) | 74.9 (74.8, 75.0) | 26.6 (26.6, 26.7) | 4.74 (4.72, 4.76) | 1.46 (1.45, 1.47) | 4.84 (4.82, 4.86) |
| 1 | 40y | 122.1 (122.0, 122.2) | 76.9 (76.8, 77.0) | 28.7 (28.6, 28.8) | 4.85 (4.82, 4.87) | 1.50 (1.49, 1.51) | 5.03 (5.01, 5.06) |
| 1 | 50y | 125.8 (125.5, 126.1) | 79.2 (79.0, 79.4) | 30.7 (30.6, 30.8) | 4.98 (4.94, 5.02) | 1.56 (1.54, 1.58) | 5.35 (5.30, 5.40) |
| 2 | 20y | 119.1 (118.9, 119.3) | 74.0 (73.8, 74.1) | 24.5 (24.4, 24.6) | 4.78 (4.73, 4.82) | 1.38 (1.36, 1.40) | 5.01 (4.97, 5.04) |
| 2 | 30y | 122.9 (122.7, 123.1) | 77.7 (77.5, 77.8) | 28.1 (28.0, 28.2) | 4.82 (4.79, 4.86) | 1.40 (1.38, 1.41) | 5.09 (5.06, 5.12) |
| 2 | 40y | 125.7 (125.4, 125.9) | 79.5 (79.3, 79.7) | 30.3 (30.2, 30.4) | 4.89 (4.86, 4.93) | 1.43 (1.42, 1.45) | 5.26 (5.22, 5.30) |
| 2 | 50y | 129.1 (128.6, 129.6) | 81.1 (80.8, 81.5) | 32.4 (32.2, 32.6) | 4.98 (4.92, 5.05) | 1.49 (1.46, 1.52) | 5.53 (5.45, 5.62) |
| 3+ | 20y | 121.9 (121.4, 122.3) | 75.8 (75.5, 76.1) | 25.2 (25.0, 25.5) | 4.90 (4.82, 4.98) | 1.36 (1.33, 1.39) | 5.15 (5.08, 5.22) |
| 3+ | 30y | 126.4 (126.0, 126.7) | 80.2 (80.0, 80.5) | 29.2 (29.0, 29.5) | 4.93 (4.87, 4.98) | 1.38 (1.36, 1.41) | 5.33 (5.28, 5.38) |
| 3+ | 40y | 128.3 (127.9, 128.8) | 81.5 (81.2, 81.8) | 31.3 (31.1, 31.5) | 4.97 (4.91, 5.02) | 1.44 (1.41, 1.46) | 5.38 (5.31, 5.44) |
| 3+ | 50y | 129.8 (128.9, 130.6) | 81.7 (81.1, 82.3) | 33.0 (32.6, 33.3) | 5.01 (4.91, 5.12) | 1.52 (1.48, 1.57) | 5.62 (5.48, 5.77) |

1 Time (years) relative to start of first pregnancy. A time of zero represents time immediately prior to conception. Predictions are at average levels of the included covariates: 27 years at first pregnancy, middle deprivation quintile, white ethnicity, no medications, singleton pregnancy, and parity of 2.

**Figure S1 Trajectories of cardiometabolic risk factors by HDP, with age on the x-axis**





**Figure S2 Trajectories of cardiometabolic risk factors by gestational diabetes, with age on the x-axis**





**Figure S3 Trajectories of cardiometabolic risk factors by multiple miscarriages, with age on the x-axis**





**Figure S4 Trajectories of cardiometabolic risk factors by stillbirth, with age on the x-axis**





**Figure S5 Trajectories of cardiometabolic risk factors by preterm delivery, with age on the x-axis**





**Figure S6 Trajectories of cardiometabolic risk factors by small for gestational age, with age on the x-axis**





**Figure S7 Trajectories of cardiometabolic risk factors by large for gestational age, with age on the x-axis**





**Figure S8 Trajectories of cardiometabolic risk factors by miscarriage, with age on the x-axis**





**Figure S9 Trajectories of cardiometabolic risk factors by major congenital anomalies, with age on the x-axis**





**Figure S10 Trajectories of cardiometabolic risk factors by any adverse pregnancy outcome, with age on the x-axis**





**Figure S11 Trajectories of cardiometabolic risk factors by number of adverse pregnancy outcomes, with age on the x-axis**





**Sensitivity analysis for those with a measure before and after pregnancy**

**Figure S12** **Trajectories of cardiometabolic risk factors by HDP, for women with a cardiometabolic measurement before and after pregnancy**





**Figure S13 Trajectories of cardiometabolic risk factors by gestational diabetes, for women with a cardiometabolic measurement before and after pregnancy**





**Figure S14 Trajectories of cardiometabolic risk factors by multiple miscarriage, for women with a cardiometabolic measurement before and after pregnancy**





**Figure S15 Trajectories of cardiometabolic risk factors by stillbirth, for women with a cardiometabolic measurement before and after pregnancy**





**Figure S16 Trajectories of cardiometabolic risk factors by preterm delivery, for women with a cardiometabolic measurement before and after pregnancy**





**Figure S17 Trajectories of cardiometabolic risk factors by small for gestational age, for women with a cardiometabolic measurement before and after pregnancy**





**Figure S18 Trajectories of cardiometabolic risk factors by large for gestational age, for women with a cardiometabolic measurement before and after pregnancy**





**Figure S19 Trajectories of cardiometabolic risk factors by miscarriage, for women with a cardiometabolic measurement before and after pregnancy**





**Figure S20 Trajectories of cardiometabolic risk factors by congenital anomalies, for women with a cardiometabolic measurement before and after pregnancy**





**Figure S21 Trajectories of cardiometabolic risk factors by any APO, for women with a cardiometabolic measurement before and after pregnancy**





**Figure S22 Trajectories of cardiometabolic risk factors by number of APOs, for women with a cardiometabolic measurement before and after pregnancy**





**Sensitivity analysis using random intercepts and random slopes for the systolic blood pressure outcome**

**Figure S23a Sensitivity analysis for random intercepts and random slopes for systolic blood pressure, trajectories by APO**





**Figure S23b Sensitivity analysis for random intercepts and random slopes for systolic blood pressure, trajectories by APO**
